# The critical role of the basal ganglia in post-stroke fatigue: A pilot study

**DOI:** 10.1016/j.ynirp.2025.100313

**Published:** 2025-12-18

**Authors:** Olga Boukrina, John DeLuca, Glenn R. Wylie

**Affiliations:** a Stroke Rehabilitation Research, Kessler Foundation, 1199 Pleasant Valley Way, West Orange, NJ, 07052, USA; b Rocco Ortenzio Neuroimaging Center, Kessler Foundation, 1199 Pleasant Valley Way, West Orange, NJ, 07052, USA; c Department of Physical Medicine and Rehabilitation, Rutgers, New Jersey Medical School, Newark, NJ, 07103, USA; d Department of Neurology, Rutgers, New Jersey Medical School, Newark, NJ, 07103, USA

**Keywords:** Stroke, Fatigue, fMRI, Basal ganglia

## Abstract

This pilot study aimed to explore the neural mechanisms underlying cognitive fatigue (CF) in stroke survivors, with a focus on the role of basal ganglia damage. Sixteen participants were recruited, including six stroke survivors with basal ganglia lesions (BG+), four with lesions elsewhere in the brain (BG−), and six healthy controls (HC). Participants underwent neuropsychological assessments and an fMRI fatigue induction task, where CF was induced using a modified letter-comparison task under individually titrated response deadlines. CF was assessed using the Visual Analog Scale of Fatigue (VAS-F), and fMRI data were analyzed to identify task-evoked activation within the fatigue network. Stroke survivors reported higher levels of CF compared to healthy controls, with the BG+ group exhibiting the highest fatigue levels and the greatest increase in fatigue over time. The BG+ group also demonstrated the most pronounced disparity in reaction times between short and long response deadlines. Functional neuroimaging revealed that CF ratings correlated with task-evoked activation in the fatigue network, but only in the BG− group. Our findings suggest that while stroke presence generally may increase CF, damage specifically involving the basal ganglia accelerates its accrual. Moreover, the ability to engage the fatigue network may mitigate fatigue, as observed in the BG− group. These results underscore the importance of basal ganglia in understanding CF and highlight the need for future research in this area.

## Highlights

• Basal ganglia stroke leads to higher cognitive fatigue (CF) and faster fatigue accrual.
• Activation in the fatigue network relates to CF in non-basal ganglia strokes.
• Brain activation differences by lesion location stress the role of basal ganglia in reducing CF.

## 1 Introduction

Neurological deficits are a debilitating consequence of stroke, which affects 795,000 Americans annually. Among these is cognitive fatigue, defined as a sense of weariness, lack of energy, and aversion to effort during mental activity. Cognitive fatigue (CF) is common following stroke with prevalence estimates varying between 29 % and 78 % of stroke survivors, depending on time of assessment (acute, subacute, and chronic stroke), diagnosis (unilateral stroke, multiple strokes, and lacunar infarcts) and the instrument used (e.g., self-report instruments, cognitive assessment batteries) (Acciarresi et al., 2014; van Heugten and Wilson, 2021; Tang et al., 2010; Van Zandvoort et al., 1998). According to a recent systematic review, post-stroke CF has a negative impact on participation in therapeutic activities during rehabilitation. Post-stroke CF is also associated with increased prevalence of anxiety and depression (Mutai et al., 2017; Pedersen et al., 2022; S. et al., 2014; Tang et al., 2010, 2013). Patients with post-stroke fatigue have worse neurological recovery and greater mortality (Hinkle et al., 2017).

Given the negative impact of CF on stroke outcomes, an improved understanding of the neural mechanisms of CF is imperative in efforts to improve stroke recovery. However, the underlying causes of post-stroke CF are currently not well understood. Some studies have reported greater incidence of CF following basal ganglia infarcts, in particular, those affecting the caudate nucleus. For example, Tang and colleagues (Tang et al., 2010) studied MRI scans of 334 acute stroke patients who were tested for post-stroke fatigue at 3 months post stroke. Among patients in the fatigue group (N = 78) the odds of having a stroke in the basal ganglia were 2.1 compared to patients in the non-fatigue group. In a follow-up study of 500 acute stroke patients, Tang and colleagues (Tang et al., 2013) reported that those with post-stroke fatigue (N = 125) had 6.4 times the odds of having a stroke in the caudate nucleus compared to patients without post-stroke fatigue. Lesions in the nearby internal capsule, thalamus, and brainstem, as well as right-sided lesions have also been linked with post-stroke CF (Hinkle et al., 2017; Mutai et al., 2017). These medial brain areas, including the basal ganglia, are frequently damaged in middle cerebral artery (MCA) strokes, which comprise 70–80 % of all strokes. However, other studies have failed to find a specific lesion location associated with fatigue (Hinkle et al., 2017).

Building on our previous work in clinical populations with traumatic brain injury (TBI) (Malloy et al., 2021; Wylie et al., 2017a) and multiple sclerosis (MS) (Genova et al., 2013; Román et al., 2022b, Román et al., 2022a; Román et al., 2022), which linked the functioning of the basal ganglia with CF, we proposed that the BG may play a central role in a distributed fatigue network. Prior neuroimaging studies support this framework, showing both structural and functional impairments in the basal ganglia and prefrontal cortex in clinical populations experiencing fatigue (Dobryakova et al., 2015; Wylie et al., 2023a). In this context, the basal ganglia are posited to maintain the balance between effort and reward (Dobryakova et al., 2013; Müller and Apps, 2019). Damage to this region may disrupt the evaluation of task demands and anticipated outcomes, making cognitive tasks feel disproportionately effortful and less rewarding. This imbalance could accelerate the accumulation of subjective CF, leading to disengagement and reduced motivation. Our earlier research has also characterized CF as arising from disruptions in a broader "fatigue network". This network includes the basal ganglia, thalamus, ventro-medial prefrontal cortex (vmPFC), dorsal anterior cingulate cortex (dACC), anterior insula, and dorsolateral prefrontal cortex (DLPFC) (Wylie et al., 2020). While the involvement of some of these areas (vmPFC, dACC, anterior insula, and DLPFC) and their susceptibility to CF seems to vary among different clinical populations, the basal ganglia and thalamus have consistently shown a response to CF across specific participant groups (Wylie et al., 2023a). Wylie et al., (2023b) studied CF in 31 individuals with MS, 31 individuals with TBI and 30 healthy HC using an fMRI fatigue induction task. During the task, participants rated their levels of CF using a visual analog scale. The results showed a positive correlation between CF ratings and activity in the caudate nucleus and a negative correlation between CF ratings and activity in the thalamus across all participants, regardless of group. Moreover, the caudate nucleus' activity varied between groups in accordance with their reported CF levels, with those experiencing higher CF exhibiting greater caudate activation as fatigue increased. This finding suggests a potential link between the caudate nucleus and the subjective experience of CF, suggesting that caudate activity may reflect the accumulation of fatigue over time. However, in this prior work, the groups studied had diffuse lesions throughout the brain rather than precisely defined lesions that did or did not include the basal ganglia. It is currently not known if participants with stroke, who tend to have high incidence of CF as well as high probability of lesions in the basal ganglia, would show comparable relationships between CF and activation in the thalamus and the basal ganglia.

The current pilot study is one of the first fMRI investigations of the neural mechanisms of post-stroke cognitive fatigue. The aim of the study was to determine the role of basal ganglia damage in subjective experience of fatigue among chronic unilateral stroke survivors. To this end we recruited three groups of participants: stroke survivors with lesions of the basal ganglia (BG+), stroke survivors with lesions elsewhere in the brain (BG−), and healthy controls (HC). Measuring CF presents unique challenges, as traditional self-report instruments such as the Fatigue Severity Scale (FSS) and the Modified Fatigue Impact Scale (MFIS) assess fatigue over extended periods and are susceptible to recall bias, mood, and other psychological factors. Objective measures, such as reaction time (RT) and accuracy, have been explored as potential indicators of fatigue, however their inconsistent relationship with subjective fatigue ratings raises concerns about their validity (Deluca, 2005). Given these challenges, we employed a real-time, task-based measure—the Visual Analog Scale of Fatigue (VAS-F)—to capture state CF dynamically and minimize these limitations.

We, additionally, aimed to investigate the impact of basal ganglia damage on activity of the “fatigue network” by directly inducing CF in the fMRI scanner while measuring task-evoked activation. We hypothesized that self-reported CF and its change with time on task would be the highest amongst those who have a stroke in the basal ganglia, less in stroke survivors with damage elsewhere in the brain, and lowest amongst healthy participants. We also expected the self-reported CF ratings to correlate with task-evoked activation in the fatigue network. This pilot study is the first step towards a greater understanding of the neural mechanisms of post-stroke CF with the potential to pave the way for new treatments that will better address this debilitating condition.

## 2 Methods

### 2.1 Participants

This study was approved by the Kessler Foundation Institutional Review Board (IRB protocol number: R-1160-21, initial approval date: August 31, 2022). We recruited 16 participants between the ages of 18 and 85 years who had English as their primary language, representing 3 groups: 6 individuals who sustained a stroke >6 months prior to study recruitment in the basal ganglia (BG+), 4 individuals who sustained a stroke >6 months prior to study recruitment elsewhere in the brain (BG−), and 6 matched healthy controls (HC) (see Table 1). Participants were excluded if they were left-handed, had a history of neurologic injury (other than a single unilateral stroke), psychiatric illness, or learning/reading disability, if they had claustrophobia, were unable to lie flat on their back, or had other limitations that would prevent participating in an fMRI study.

**Table 1:** Demographics of the 3 participant groups. Groups were evenly matched for age, educational attainment, and sex distribution.

	BG+ (N = 6)	BG− (N = 4)	HC (N = 6)	Overall (N = 16)
**Age**				
Mean (SD)	57.7 (15.7)	55.3 (11.0)	62.8 (13.3)	59 (13.3)
Median [Min, Max]	58.5 [30.0, 74.0]	56.0 [42.0, 67.0]	67.5 [36.0, 71.0]	65.0 [30.0, 74.0]
**Education level**				
GED	3 (50.0 %)	0 (0 %)	1 (16.7 %)	4 (25.0 %)
Bachelors	2 (33.3 %)	2 (50.0 %)	3 (50.0 %)	7 (43.8 %)
Masters	1 (16.7 %)	2 (50.0 %)	2 (33.3 %)	5 (31.3 %)
**Sex**				
Female	4 (66.7 %)	1 (25.0 %)	2 (33.3 %)	7 (43.8 %)
Male	2 (33.3 %)	3 (75.0 %)	4 (66.7 %)	9 (56.3 %)

### 2.2 Materials

Validated neuropsychological tests were used to assess learning, memory, mood, sleep, post-stroke cognition, state, and trait cognitive fatigue, as follows.

Learning and Memory: Participants completed the Florida Mental Status Examination (FMSE) (Doty et al., 1990) digit span task, California Verbal Learning Test, 2nd edition (CVLT-II), and Montreal Cognitive Assessment (MoCA) (Pendlebury et al., 2015).

Mood, Sleep, and Motivation: Depression was assessed with the Beck Depression Inventory (BDI) (Beck et al., 1961) and anxiety was assessed using the State Trait Anxiety Inventory (STAI) (Spielberger and Barratt, 1972). Sleep quality was measured with the Pittsburgh Sleep Quality Index (PSQI) (Buysse et al., 1989). Motivation was assessed with the Behavioral Inhibition, Behavioral Activation scales (BIS/BAS) (Carver and White, 1994).

Post-stroke Cognitive Deficits: Additionally, considering that unilateral stroke is often associated with spatial neglect, which is characterized by asymmetric performance between the left and right hemispace, we assessed spatial neglect using the Apples Test and a letter recognition task from the Reading Comprehension Battery for Aphasia (RCBA) (Flanagan and Jackson, 1997).

**Fig. 1:**
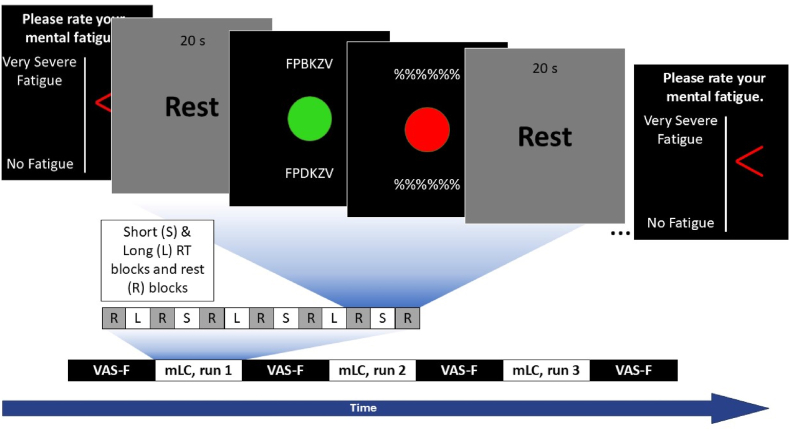
MLC task design. Each run of mLC consisted of 20 s of rest, with alternating short (n = 3) and long (n = 3) response window blocks, with a final rest period at the end of the run. Cognitive fatigue was measured at the start and after each run of mLC.

State CF Assessment: State CF was assessed during the performance of the fMRI task (see below) using a visual analog scale of fatigue (VAS-F) (Lee et al., 1990). This scale ranges from no fatigue to maximal fatigue (see Fig. 1). The VAS-F provides a momentary, real-time assessment of fatigue, which helps mitigate the limitations of trait fatigue measures by capturing fluctuations in CF as they occur. By interpreting VAS-F in the context of behavioral and fMRI data, we sought to establish a more comprehensive and reliable approach to measuring CF.

Trait CF Assessment: Trait CF was assessed with the Modified Fatigue Impact Scale (MFIS) (Flachenecker et al., 2002; Kos et al., 2005; Vickrey et al., 1995), a 21-item questionnaire providing an assessment of the effects of fatigue in terms of physical, cognitive, and psychosocial functioning. The MFIS score ranges from 0 to 84, with higher scores representing higher levels of fatigue; a score >38 defines significant fatigue.

### 2.3 fMRI tasks, instrumentation, and acquisition parameters

Fatigue-Inducing fMRI Task: A letter-comparison task was used to induce CF while participants were in the fMRI scanner. This was a version of the Letter Comparison task (Salthouse, 1996) that was modified in several ways to accommodate the restrictions of the MRI environment and the studied population (Genova et al., 2019). On every trial of this task, participants were presented with two strings of letters, one above the other, at the center of the screen. This was a modification from the original horizontal layout to account for performance differences in left and right space among patients with stroke who may have symptoms of spatial neglect. On some trials, the letter strings were identical; on other trials, the letter strings differed by one letter. The participants were asked to press one button if the letter strings were the same and another button if they were different. An equal number of same and different letter strings were presented, and (in the case of differing strings) the location of the difference was equiprobable within the string. The letter strings randomly varied in length from three to nine characters long. Stimuli were presented using ePrime software (Version 3.0.3.80) that recorded participants' response times and accuracy. To induce fatigue, participants completed three ∼9.5 min runs of the letter-comparison task.

The difficulty of the mLC task was manipulated using a deadline procedure. In ‘Short’ response window blocks, the deadline was short; in ‘Long’ response window blocks, the deadline was long. The length of the response window was individually determined for each participant, based on participants' performance while practicing the task. Prior to scanning, each participant practiced the task until their accuracy reached 80 % correct. When this criterion was reached, the mean response time (RT) was calculated from the most recent block and was used to determine the deadlines used during scanning: the stimuli were presented for 80 % of each participant's mean RT for the short response window blocks and for 120 % for the long response window blocks. Once the deadline period was reached, the stimuli were masked with a string of “%” characters that were matched in length to the letter strings used on each trial. The stimuli and masks were presented for a total of 4 s, while the specific amount of time each was presented was determined by each participant's mean RT. This approach ensured that all participants were presented with the same amount of stimulation and the same number of trials, but that the stimulus processing deadline was individually titrated.

Inside the scanner, each participant worked through three runs of the mLC task. Each run began with an instruction (2 s) that was either “Short” or “Long”. Thereafter, there was a 20 s block of rest before participants alternated regularly between blocks with short and long response windows (i.e., deadlines), interleaved with rest blocks (i.e., Rest, Short, Rest, Long, Rest, etc.), with three short blocks and three long blocks per run (see Fig. 1). Within each block, participants performed eight letter-comparison trials. The fMRI data were modeled with a box-car block-design, in which there were nine observations of the short and long blocks per participant across the 3 fMRI runs.

State CF Assessment: Before and after every run, participants were asked to rate their perceived level of CF assessed using the VAS-F. Participants were instructed to rate the CF they were experiencing “right now, at this moment”. The ratings were acquired using a response box: one button moved a cursor up and the other button moved the cursor down along the vertical line with a starting position at the beginning of the line (No Fatigue). The vertical position of the cursor was converted to a percentage of the height of the scale, and this was used as the participants’ CF rating.

Magnetic Resonance Imaging Acquisition: MRI data were obtained using a 3.0 T S Skyra scanner equipped with a 20-channel head coil. All participants were instructed to minimize head motion. Foam padding was used for additional movement restriction.

Structural imaging: Each scan session began with the acquisition of high-resolution anatomical images using Magnetization-Prepared Rapid Gradient-Echo sequence (MP-RAGE). This 3D isotropic sequence was acquired sagittally (TR = 2100 ms, TE = 3.43 ms, Flip angle = 9°, effective TI = 900 ms, 256x256 matrix size, FOV = 256 mm, 1 mm slice thickness, 176 slices to cover the whole brain, total imaging time = 5m 38s). The MP-RAGE, along with the fieldmap data, was used to calculate the warping field used to warp the functional data into standard space. In addition, a T2-weighted Fluid Attenuated Inversion Recovery (FLAIR) scan was acquired (TR = 9000 ms, TE = 91 ms, 50 slices, 1 × 1 × 3 mm^3^ voxels) and was used along with the MP-RAGE for lesion mapping.

Fieldmaps: A fieldmap with 36 axial slices that covered the whole brain was acquired using a dual echo sequence with the following parameters: TR = 400 ms, TE = 4.92/7.38 ms, FOV = 220 mm, slice thickness = 3 mm, gap = 1 mm, matrix size = 92x92, flip angle = 40°. The calculated field map was used to correct the functional images.

Functional MRI: Functional neuroimaging was performed using rapid multi-slice gradient echo, T2∗-weighted echo-planar (EPI) images (TR = 1.5s, TE = 30ms, 44 slices, gap = .5 mm, 2 mm^3^ isotropic voxels). The pitch of the slices was increased by 30° from the AC-PC line to optimize the acquisition of the orbital cortex and to ensure that the cerebellum was included in the field of view (Deichmann et al., 2003). For each of the blocks of task-related data, 380 vol were acquired, for a total scanning time of ∼9.5 min per run.

### 2.4 Procedure

HCs were recruited from the community. Stroke participants were recruited through the Kessler Institute for Rehabilitation hospital admissions records accessed with prior patient approval in accordance with institutional and HIPAA rules. Eligible participants were screened via the telephone. Participants who indicated research interest and met the inclusion criteria were asked to complete the informed consent and to take part in the study. All participants completed the neuropsychological assessment and underwent a neuroimaging session that included the acquisition of MRI and fMRI data. The neuropsychological assessment data were collected to characterize the sample and to identify potential confounds that differed between groups (e.g., depression). Testing took approximately 2 h with built-in rest breaks.

### 2.5 Analysis

Lesion mapping: Lesions were labeled using a combination of manual segmentation and automated intensity-based voxel selection. T1-weighted and T2 FLAIR images were overlaid onto each other to assist in identification of voxels with abnormal intensity. To avoid warping of the lesion area during registration to the anatomical template, we applied cost-function masking of the input image using the inverse of the lesion mask (lesion weights). Lesion volume, normalized by total brain volume, was used as a covariate in the analyses.

Spatial Normalization and Localization of Functional Activity: The fMRI data were preprocessed using fMRIPrep version 1.4.1 (Esteban et al., 2019). Preprocessing steps included slice timing correction, spatial realignment, co-registration to the T1 MPRAGE image for localization of activated areas, nonlinear warping into standard MNI space, estimation of confounds such as framewise displacement (FD), DVARS (Power et al., 2014) and global signals associated with white matter, cerebrospinal fluid and a mask of the whole-brain. Additionally, a set of physiological regressors was extracted to allow for component-based noise correction (*CompCor* (Behzadi et al., 2007)). The data were smoothed (6 mm FWHM) to minimize anatomical differences and increase the signal to noise ratio, then each voxel was scaled to the grand mean intensity of that voxel across the acquisition run. A deformation field to correct for susceptibility distortions was estimated based on the field map that was co-registered to the BOLD reference, using a custom workflow of fMRIprep and further improvements of HCP pipelines (Glasser et al., 2013).

In all cases, the data were checked for excessive motion (a shift of more than 3.5 mm, or 1° of angular motion). Data acquisition runs with excessive motion were discarded. Individual acquisitions in which movement exceeded 0.5 mm in Euclidian distance were excluded from further analysis using the ‘censorTR’ function in 3dDeconvolve. In all cases, motion parameters and their derivatives from the realignment step were used as regressors of no interest in the deconvolution. In addition, FD and the first six components from aCompCor were included as regressors of no interest. CF was modeled using an amplitude modulated regressor (e.g., Motes et al., 2017) in the 3dDeconvolve program. This approach uses a regressor of unit amplitude to model the response to the task and a regressor of varying amplitude to model the condition of interest – in our case, CF (see Fig. S1 in the Supplementary Materials for an example). The amplitude of the second regressor was the mean of the CF score reported before and after each run. All three runs of the mLC task were modeled in a single deconvolution for each participant, resulting in four beta weights: one for the task when the response window was long and one when it was short, one beta weight for CF when the response window was long and one when it was short. These beta weights were used in the group-level analyses.

Group Analyses: For the demographic data, the categorical variables of sex and educational attainment were analyzed with a χ^2^ test. For both analyses, one factor was Group (BG+, BG−, Control); for the analysis of sex the other factor was Sex (male, female); for the analysis of educational attainment, the other factor was Education (as degree: GED, bachelor's degree, master's degree, or as years of education). Age and scores on the neuropsychological tests were analyzed using a one-way ANOVA, with the factor of Group (as above).

The VAS-F data were analyzed using a linear mixed effects (LME) model with the following factors: Group (as above), CF Rating Number (rating 0–3), and Lesion Volume; participant was a random factor. The rate of accrual of CF – cognitive fatigue rate (CFR) – was calculated by fitting a regression line to the VAS-F scores for each participant (across the three runs of the mLC task), and the slope and intercept of this line were saved. The CFR data were then analyzed with an LME model with the factors of Group (as above), controlling for Lesion Volume and intercept (intercept was included in the model to account for the fact that some Stroke survivors had very high intercepts, resulting in low slope values due to ceiling effects).

The RT and accuracy data were analyzed with LME models with the factors of Group (as above), Deadline (long vs short response window), VAS-F (state CF, entered as a quantitative variable), Run (runs 1–3) and Lesion Volume (included to account for stroke severity). Because the VAS-F scores were skewed, they were transformed using a Box-Cox transform. For RT, only RTs from correct trials were included in the analysis. Moreover, RT and accuracy data from each block were only included if accuracy for that block was greater than 50 %. Considering the modest group sizes, we report effect sizes (ES, Partial *η*^*2*^ and Cohen's d) for all observed effects that were part of our *a priori* hypotheses.

The neuroimaging data were analyzed with an LME model (3dLMEr from the AFNI suite of processing tools) with the factors of Group (as above), Deadline (as above), and Lesion Volume (as above). This model was applied to both the task-related data (modeled with unit amplitude regressors) and the CF-related data (modeled with amplitude modulated regressors). Planned comparisons were also calculated, in which brain activation (for the task and for CF) was assessed for each group, collapsing across response window. Only runs resulting in CF were included in the analysis (i.e., runs in which the VAS-F score was greater than zero). This resulted in the exclusion of ∼7 % of runs (for details see Table S1 in the Supplementary Materials).

The results of these analyses were corrected for multiple comparisons by using an individual voxel probability threshold of p < 0.01 and a cluster threshold of 67 voxels (voxel dimension = 2.0 x 2.0 × 2.5 mm). Monte Carlo simulations, using 3dClustSim (version AFNI_24.0.17, compile date: Mar 24, 2024) showed this combination resulted in a corrected whole brain alpha level of p < 0.05. Using the same approach, we calculated the corrected alpha level for the areas within the fatigue network (about which we had prior hypotheses) to be 30 voxels.

## 3 Results

Demographics: As Table 1 shows, the groups were matched on age, sex, and educational attainment, which was confirmed by considering both educational degree and years of education (Age: *F*(2,13) = 0.41, *p* = 0.68; Years of Education: *F*(2,13) = 2.09, *p* = 0.16; Education Level: *χ*^*2*^ = 2.0, *p* = 0.37; Sex: *χ*^*2*^ = 0.3, *p* = 0.62; Education Level x Sex: *χ*^*2*^ = 0.76, *p* = 0.68).

**Fig. 2:**
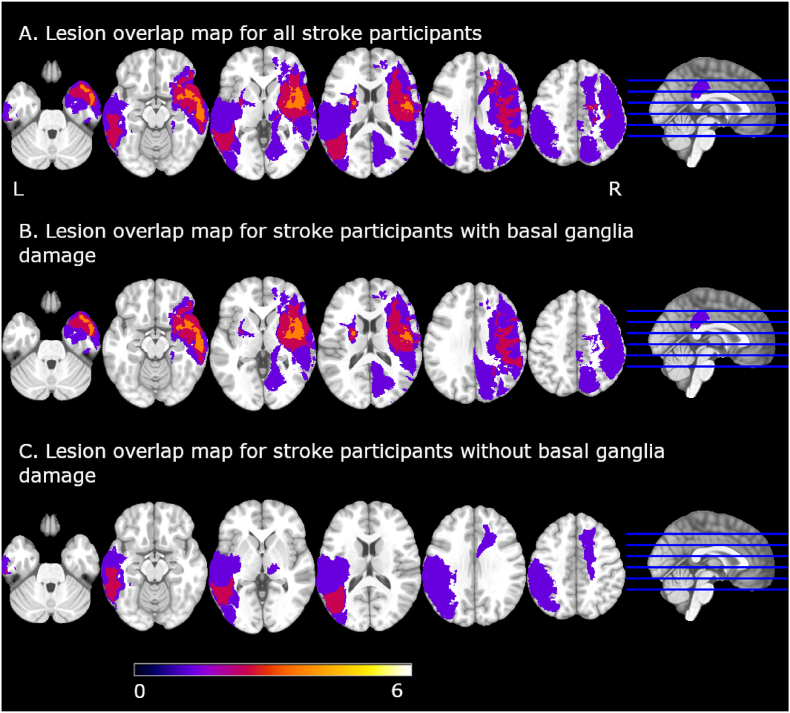
Lesion overlap map on a standard brain template (MNI152 2 mm brain). The scale is discrete and represents the number of participants with lesions in each area. Images in neurological orientation.

Lesions: Lesion maps for all stroke participants and for the BG− and the BG+ groups separately are shown in Fig. 2. Individual maps for participants in each group can be found Figs. S2 and S3.

Neuropsychological Assessment: The groups did not differ significantly on measures of depression, anxiety, sleep quality, or motivation (Table 2, all p-values >0.05). We also did not find group differences in trait CF (as measured by the MFIS) or visuospatial processing. There was a nonsignificant trend on the MOCA, where the BG+ group scored worse than HC (*F*(2,13) = 2.89, *p* = 0.09, partial *η*^*2*^ = 0.31, large effect size (ES)).

**Table 2:** Neuropsychological assessment scores for the 3 participant groups. Means and standard deviations shown.

	BG+ (N = 6)	BG− (N = 4)	HC (N = 6)	P-value
**Anxiety**				
STAI: State Anxiety (Max Score = 80)	31.3 (9.3)	30.3 (10)	28.8(5.3)	0.87
STAI: Trait Anxiety (Max Score = 80)	31.2 (10.8)	38.3 (11.3)	37.5 (15.5)	0.62
**Motivation: Behavioral Inhibition, Behavioral Activation scales**				
BAS Drive (Max score = 16)	10.2 (2.5)	12.5 (1.7)	9.7 (4)	0.35
BAS Fun Seeking (Max score = 16)	10.2 (2.8)	12.3 (1.7)	10.2 (2.8)	0.52
BAS Reward Responsiveness (Max score = 20)	16.3 (2.7)	18.3 (1.3)	16.8 (1.8)	0.40
BIS (Max score = 28)	15.5 (4.4)	16.8 (3.8)	17.3 (2.9)	0.70
**Depression**				
BDI, max score = 63; 11–16 mild, 17–20 borderline, 21–30 moderate, 31–40 severe, >40 extreme depression	9.5 (9.4)	10.5 (4.7)	7.8 (8)	0.87
**Fatigue**				
MFIS physical (Max score = 36)	13.5 (7.4)	21.5 (9)	11.2 (6.9)	0.14
MFIS cognitive (Max score = 40)	15.7 (10.5)	15.5 (9.7)	12.3 (9.2)	0.82
MFIS psychosocial (Max score = 8)	2.7 (3.2)	4.3 (2.2)	2.7 (3.1)	0.66
MFIS total (Max score = 84)	31.8 (20.6)	41.3 (19)	26.2 (17.7)	0.49
**Learning and Memory**				
Forward Digit Span (Max score = 5)	4.3 (0.5	4 (1.4)	4.3 (0.5)	0.78
MOCA (Max score = 30)	22.5 (2.1)	24.3 (2.8)	25.8 (2.5)	0.09
**Sleep Quality**				
PSQI (Max score = 21, higher scores = worse sleep quality)	5.8 (4.1)	9.3 (1.5)	5.8 (5.7)	0.44
**Visuospatial Processing**				
Apples test (Max score = 50)	40.2 (13.7)	48.3 (1.7)	49.2 (1)	0.19

*Note*: BAS – Behavioral approach scale; BIS – Behavioral Inhibition Scale; BDI – Beck Depression Inventory; MFIS – Modified Fatigue Impact Scale; MOCA – Montreal Cognitive Assessment, PSQI – Pittsburgh Sleep Quality Index, STAI – State Train Anxiety Index.

**Fig. 3:**
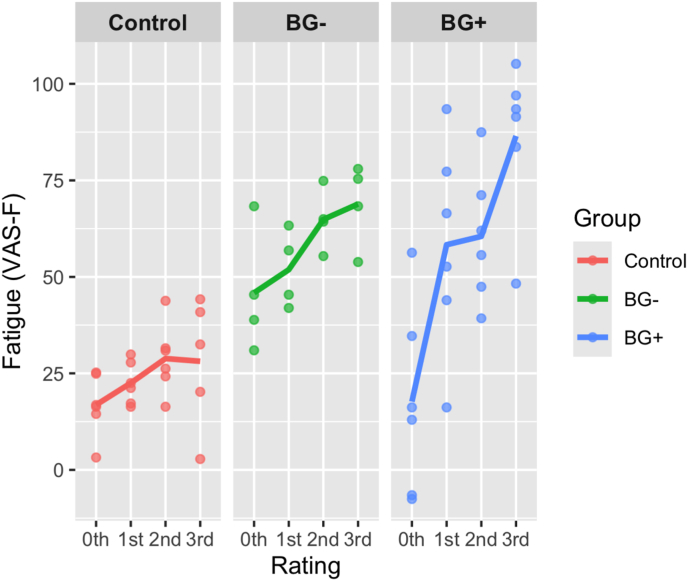
**Cognitive fatigue in the three groups across the course of the experiment.** The data from the HC are shown in red, the BG− group is shown in green and the BG+ group is shown in blue. Ratings were provided at baseline and after each of the three task blocks.

State CF (VAS-F): The main effect of Rating was significant and had a large ES (*F*(3,38.0) = 9.27, *p* < 0.001, *η*^*2*^ = 0.42). Participants reported increasing CF across the 4 ratings, with mean values for ratings 0–3 being 26.8, 44.2, 51.4, 61.2, respectively. There was also a significant Group by Rating interaction (*F*(6,38.0) = 2.67, *p* < 0.05, *η*^*2*^ = 0.30). The BG+ group showed a greater rate of CF increase across ratings than the BG− and the HC groups, while the BG− group showed a greater rate of CF increase across ratings than the HC group, as evidenced by steeper slopes (see Fig. 3). The CFR data, computed as detailed in the *Analyses* section, confirmed this interpretation, with a large and significant effect of Group (*F*(2,11) = 11.60, *p* < 0.01, *η*^*2*^ = 0.68), where the rate of CF increase in the BG+ group (CFR = 17.78) was greater than in the HC (CFR = 3.03; *t*(11) = 3.43, p < 0.05) while the difference between HC and the BG− group (CFR = 13.44) was not-significant (*t*(11) = 2.23, p = 0.11). The difference between the BG+ and BG− groups was also not significant (t(11) = 0.96), p = 0.62).

The effect of Group on CF ratings had a large ES and approached significance (*F*(2,11.86) = 3.55, *p* = 0.06, *η*^*2*^ = 0.37). Across all timepoints, the HC group reported less CF than either of the BG− or BG+ groups (HC = 24.1, BG− = 57.9, BG+ = 55.8).

**Fig. 4:**
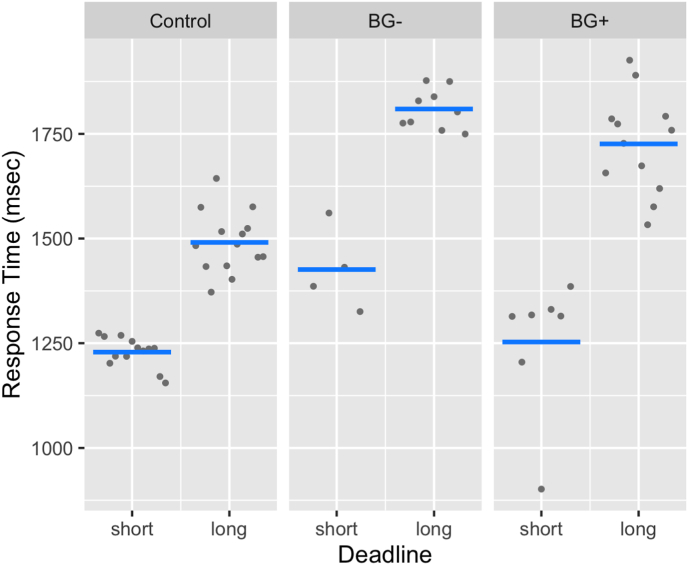
**The interaction between Group and Deadline in RT.** The RT for the two conditions (short vs. long) are shown separately for the three groups (Control, BG− and BG+).

Response Time (RT): For the RT data, the main effect of Deadline was significant (*F*(1, 37.93) = 45.85, *p* < 0.0001, η^2^ = 0.55, large ES) and resulted from participants responding with shorter latencies when they were given a short response window (M = 1348ms) than when they were given a long response window (M = 1720ms). The effect of VAS-F was significant (*F*(1, 33.14) = 6.93, *p* = 0.01, *η*^*2*^ = 0.17, medium ES) with a coefficient of −3.5. That is, for each unit increase in VAS-F, participants tended to respond 3.5 ms faster. There was also a significant interaction between Group and Deadline (*F*(2, 37.63) = 3.25, *p* < 0.05, *η*^*2*^ = 0.15, medium ES) (see Fig. 4). For the HC and the BG+ groups, the difference between short and long response windows was significant (HC: *t*(36.82) = 4.92 *p* < 0.001); BG+: *t*(36.63) = 7.47, *p* < 0.0001), while for the BG− group this difference was not (*t*(39.01) = 2.641, *p* > 0.10).

Error Rates: The error rate data were analyzed with the same model. The only significant effect was the main effect of Deadline (*F*(1, 29.88) = 5.21, *p* < 0.05, *η*^*2*^ = 0.15, *d* = 0.65). This resulted from participants responding with higher accuracy (72.7 %) during the long response window blocks and lower accuracy (64.6 %) during the short response window blocks.

Neuroimaging:

Task-related brain activation: As a manipulation check, we first analyzed the data modeled with the invariant (unit amplitude) regressor (see Fig. S1). This regressor modeled the neural response to the task. All three groups showed strong activation in the fronto-parietal network, combined with deactivation in the default-mode network (see Fig. S4). This, along with the behavioral data, showed that the participants were engaged in the task.

**Fig. 5:**
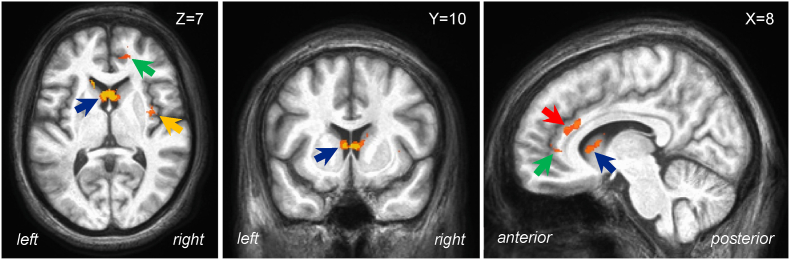
**Brain areas associated with CF in the BG**− **group.** Three orthogonal views are shown (X Y Z = 8, 10, 7). CF-related activation is shown in the caudate nucleus (blue arrow), the ACC/vmPFC (green arrow), the insula (yellow arrow) and the ACC (red arrow).

Fatigue-related brain activation: For the whole-brain CF-related brain activation, we found activation in the BG− group in four areas, all of which are thought to be within the fatigue network: the caudate nucleus, the vmPFC, the ACC and the insula (see Fig. 5 and Table 3). The same comparisons for the HC and BG+ groups did not produce any significant effects. There were also no main effects of Group or Deadline, and no interaction between Group and Deadline.

**Table 3:** **Brain areas showing significant CF-related BOLD activation in the BG**− **group.** X Y Z = the location of the voxel with peak intensity in each cluster; N Voxels refers to the number of voxels in the cluster; Z Stat refers to the maximal Z statistic in each cluster.

Location	MNI Coordinates			Number of Voxels	Z Statistic
	X	Y	Z		
Caudate Nucleus	−2	14	4	253	3.05
Anterior Cingulate Cortex/vmPFC	8	42	2	52	3.16
Anterior Cingulate Cortex	10	34	22	39	2.68
Insula	36	−2	7	31	2.81

Note: MNI – Montreal Neurological Institute; vmPFC – ventromedial prefrontal cortex.

## 4 Discussion

This pilot study is among the first to use fMRI to directly investigate the neural mechanisms underlying post-stroke CF, providing new insights beyond prior research which relied on broad lesion-symptom associations (Tang et al., 2010, 2013). While previous studies have implicated the basal ganglia lesions in post-stroke CF, our pilot study took a more targeted approach by examining the differential impact of basal ganglia versus non-basal ganglia lesions on both subjective cognitive fatigue experience and neural activation within the areas thought to be part of the fatigue network (Dobryakova et al., 2013; Wylie et al., 2017b). To address the long-standing challenge of measuring state CF objectively, we employed a momentary, quantitative, task-based rating (VAS-F) during fMRI scanning, rather than relying on retrospective instruments, which are often influenced by recall bias, mood, and other psychological factors. This methodological advancement allowed us to capture dynamic fatigue fluctuations and their direct neural correlates, bridging the gap between subjective fatigue perception and underlying brain activity.

In the fMRI fatigue induction task, we found a statistically significant interaction between group and the timing of the rating, indicating that the rate of fatigue increase over time varied across the three participant groups: stroke participants with basal ganglia damage (BG+), stroke participants with damage elsewhere in the brain (BG−) and healthy controls (HC). As illustrated in Fig. 2, individuals in the BG− group had a numerically greater rate of CF increase than the control participants. Moreover, stroke individuals in the BG+ group exhibited an even greater numeric increase in CF over time compared to those who had sustained a stroke in other brain regions or the HC group. When looking at the rate of CF accrual overtime as a function of group, we found that the BG+ group had a significantly steeper increase in CF over time on task compared to the HC group. The BG− group fell in-between the BF+ and the HC groups, but did not differ significantly from either.

These findings provide weak preliminary evidence that the presence of stroke itself increases fatigue, but strong evidence that the location of stroke lesions, particularly the involvement of the basal ganglia, influences the rate of CF accrual over time. Additionally, our results may suggest that among stroke survivors, damage to the basal ganglia makes individuals less capable of mitigating CF as time on task increases, potentially contributing to CF burden.

The fMRI fatigue induction task was administered with two different response window deadlines. Imposing a time limit (deadline) on participants' response time appeared to influence their response strategy during blocks with short response windows compared to those with longer response windows. Under the constraint of a short response window, participants across all groups demonstrated shorter reaction times but made more errors. This shift suggests that they adopted a more liberal response bias to cope with the time pressure. There was also variation among the groups in terms of the extent of RT differences between the short and long response window conditions, as well as in the variability of individually adjusted response windows around the group average. The most significant disparity between short and long response window trials was seen in the BG+ group. Their reaction times in the short response window condition were more akin to those of the healthy control group, while in the long response window condition, their RTs were closer to those of the BG− group. These RT data were interpreted in the context of greater accuracy in the long than short response window conditions. We have previously shown that CF correlates with response bias (Wylie et al., 2021). In this analysis we added another layer of nuance, by specifying how this relationship may be impacted by a response time constraint. Specifically, while CF may lead to a more conservative response bias, imposing a deadline has the opposite effect, encouraging a more liberal bias.

In the analysis of RT that incorporated VAS-F, we found that CF ratings increased as reaction times decreased. This observation aligns with cognitive computational models of fatigue, which suggest that fatigue accumulates trial-by-trial as a function of the effort expended on each trial (Matthews et al., 2023; Ulrichsen et al., 2020). In the context of our task, responding accurately within a shorter time frame necessitated greater effort, thereby leading to heightened fatigue by the end of the block. However, it contrasts with other studies that have used a fatigue induction paradigm in other populations without a response deadline (Wylie et al., 2023a). Whether this difference is related to the population studied or to the response deadline will require more research.

In this study we also tested a second hypothesis that the self-reported CF ratings would correlate with task-evoked activation in the fatigue network. This hypothesis was supported, but only for the BG− group—participants who had sustained brain damage outside of the basal ganglia, resulting in CF. In previous work, we hypothesized that the basal ganglia are a hub of the fatigue network (Wylie et al., 2023a). Therefore, it is possible that the BG− group, despite their brain damage, was able to engage the basal ganglia and the larger fatigue network to partially mitigate their CF. This contrasts with the BG+ group, who showed no significant activation in the contralesional basal ganglia or other areas of the fatigue network and reported the highest levels of CF. On the other hand, the Control group also showed no significant activation in the fatigue network but reported the lowest levels of CF. The absence of significant fatigue network activation in the HC group, combined with their low CF levels and the small sample size, likely contributed to this null effect.

The relationship between CF and brain activity in the BG− group was positive in the four areas where a significant relationship was found (see Fig. 5), including the left caudate nucleus, and the right anterior cingulate cortex, ventromedial prefrontal cortex, and insula. The activation of the caudate with increasing fatigue once again underscores the critical role of this area in the development of CF. It is also consistent with previous stroke studies, which have shown that individuals with post-stroke fatigue had 6.4 times the odds of having a stroke in the caudate nucleus compared to those without post-stroke fatigue (Tang et al., 2013). Interestingly, the positive relationship between caudate activation and CF contrasts with other findings, where caudate activation was negatively related to fatigue in healthy controls and participants with Multiple Sclerosis, but positively related in Traumatic Brain Injury participants (Wylie et al., 2023a). This suggests that the CF-related activation observed in the BG− group may be adaptive, as this group reported numerically lower CF than the BG+ group, but it is not optimal, given that they reported more CF than the HC group. Given the complexity of these results, the relationship between CF and brain activity in the areas of the fatigue network remains an important area of future study. Furthermore, given that the anterior cingulate gyrus and medial prefrontal cortex (PFC) are typically involved in emotional processing (Barbas, 2000), their activation in association with CF ratings could indicate that cognitive fatigue may be mediated by an increased demand on these regions. This could suggest a mechanism where, under conditions of fatigue, the brain shifts resources towards the medial PFC and anterior cingulate to manage the emotional and cognitive burden. This contrasts with the typical deactivation observed during high cognitive load (Habeck et al., 2005), indicating that fatigue might trigger a compensatory response in these regions to maintain cognitive and emotional balance. This mechanism may also reflect integration of the emotional and cognitive aspects of fatigue, potentially leading to the subjective experience of exhaustion. Additionally, activation near our insular peak was previously linked to goal-directed behavior (Laurens et al., 2005). This may indicate that this region plays a role in modulating engagement during cognitively demanding tasks, such as the one employed in this study.

The areas activated in our study have also been postulated to play a role in the allocation of control during mentally effortful tasks. According to the Expected Value of Control (EVC) theory, the brain evaluates whether to engage control by integrating information about anticipated rewards, associated effort costs, and the likelihood of successful task performance (Shenhav et al., 2025; Shenhav et al., 2013). Within this framework, the dorsal part of the ACC is proposed to play a central role in specifying the amount of control to allocate, while the value signals themselves (e.g., reward, effort costs, task goals) are generated in other regions, including the basal ganglia, ventromedial PFC, and insula, before being relayed to the dorsal ACC. Our results, which implicate these regions in the subjective experience of CF, support the notion that disruptions in value computation (e.g., from BG damage) or in the monitoring and implementation of control (e.g., via dACC or vmPFC) may underlie fatigue. Specifically, in the BG− group, activity in the caudate may reflect effort-related value representation used to guide sustained engagement, whereas in the BG+ group, the lack of such recruitment may lead to inefficient or dysregulated allocation of control, resulting in steeper CF accumulation. This interpretation underscores the relevance of EVC theory as a framework for understanding fatigue as a failure in cost-benefit integration across distributed brain systems.

Given the complexity of the observed relationships between CF and the involvement of the basal ganglia, future research should aim to refine our understanding of how different neural mechanisms contribute to fatigue regulation in stroke survivors who often sustain brain damage affecting midline brain structures. A critical next step is to increase sample size to determine whether the observed fatigue-related activation patterns remain stable. Additionally, future studies should explore the relationship between the rate of CF accrual with time on task and participation in post-stroke rehabilitation, as this may help develop personalized intervention strategies, with the potential to improve outcomes for stroke survivors experiencing persistent cognitive fatigue.

## 5 Conclusions

Our study highlights the critical role of brain lesion location, particularly in the basal ganglia, in influencing cognitive fatigue (CF) among stroke survivors. While stroke may itself increase fatigue, damage to the basal ganglia accelerates its accumulation over time. Stroke survivors with basal ganglia damage showed the highest CF levels and the greatest fatigue increase, suggesting reduced ability to engage the fatigue network effectively. Additionally, the relationship between CF and brain activation varied by lesion location. In the BG− group, positive activation in areas such as the left caudate nucleus, the right anterior cingulate cortex, ventromedial prefrontal cortex, and insula was associated with CF. Combined with behavioral data, this suggests that intact basal ganglia function may help mitigate fatigue, albeit not optimally. This activation pattern may reflect a compensatory mechanism where the brain reallocates resources to manage the cognitive and emotional burden of fatigue, a process that appears to be less effective in those with basal ganglia damage. Future work in this area should focus on understanding the compensatory mechanisms within the fatigue network and how they differ based on lesion location, while also increasing the sample size and replicating the current pilot results. Future studies should also explore the impact of targeted interventions, such as cognitive training, neuromodulation, or aerobic exercise, on fatigue network activation and CF accrual, with the goal of developing personalized treatment strategies to improve functional outcomes for stroke survivors experiencing persistent and progressively worsening CF.

## CRediT authorship contribution statement

**Olga Boukrina:** Writing – review & editing, Writing – original draft, Visualization, Validation, Supervision, Project administration, Methodology, Investigation, Formal analysis, Data curation, Conceptualization. **John DeLuca:** Writing – review & editing, Validation, Methodology, Conceptualization. **Glenn R. Wylie:** Writing – review & editing, Writing – original draft, Visualization, Validation, Supervision, Software, Resources, Project administration, Methodology, Investigation, Funding acquisition, Formal analysis, Data curation, Conceptualization.

## Declaration of generative AI and AI-assisted technologies in the writing process

During the preparation of this work the authors used ChatGPT in order to improve readability of the manuscript. After using this tool, the authors reviewed and edited the content and take full responsibility for the content of the published article.

## Funding sources

This work was supported by the pilot grant from the David F. Bolger Trust to G. Wylie (PI).

## Declaration of competing interest

The authors declare that they have no known competing financial interests or personal relationships that could have appeared to influence the work reported in this paper.

## Data Availability

Data will be made available on request.

## References

[bib1] Acciarresi Monica, Bogousslavsky Julien, Paciaroni Maurizio (2014). Post-Stroke fatigue: epidemiology, clinical characteristics and treatment. Eur. Neurol..

[bib2] Barbas Helen (2000). Connections underlying the synthesis of cognition, memory, and emotion in primate prefrontal cortices. Brain Res. Bull..

[bib3] Beck A.T., Ward C.H., Mendelson M., Mock J., Ernbaugh J. (1961). An inventory for measuring depression. Arch. Gen. Psychiatry.

[bib4] Behzadi Yashar, Restom Khaled, Liau Joy, Liu Thomas T. (2007). A component based noise correction method (CompCor) for BOLD and perfusion based FMRI. Neuroimage.

[bib5] Buysse D.J., Reynolds C.F., Monk T.H., Berman S.R., Kupfer D.J. (1989). The Pittsburgh sleep quality index: a new instrument for psychiatric practice and research. Psychiatry Res..

[bib6] Carver C.S., White T.L. (1994). The BIS_BAS Scales.Pdf. J. Personality Soc. Psychol..

[bib7] Deichmann R., Gottfried J.A., Hutton C., Turner R. (2003). Optimized EPI for FMRI studies of the orbitofrontal cortex. Neuroimage.

[bib8] Deluca John (2005). Fatigue as a Window to the Brain.

[bib9] Dobryakova Ekaterina, DeLuca John, Genova Helen M., Wylie Glenn R. (2013). Neural correlates of cognitive fatigue: Cortico-Striatal circuitry and effort–reward imbalance. J. Int. Neuropsychol. Soc..

[bib10] Dobryakova Ekaterina, Genova Helen M., DeLuca John, Wylie Glenn R. (2015). The dopamine imbalance Hypothesis of fatigue in multiple sclerosis and other neurological disorders. Front. Neurol..

[bib11] Doty L.C., Bowers D., Heilman K.M. (1990). Florida mental status exam for progressive dementia screening. Gerontol..

[bib12] Esteban Oscar, Markiewicz Christopher J., Blair Ross W., Moodie Craig A., Ilkay Isik A., Erramuzpe Asier, Kent James D., Goncalves Mathias, DuPre Elizabeth, Snyder Madeleine, Oya Hiroyuki, Ghosh Satrajit S., Wright Jessey, Durnez Joke, Poldrack Russell A., Gorgolewski Krzysztof J. (2019). FMRIPrep: a robust preprocessing pipeline for functional MRI. Nat. Methods.

[bib13] Flachenecker P., Kümpfel T., Kallmann B., Gottschalk M., Grauer O., Rieckmann P., Trenkwalder C., Toyka K.V. (2002). Fatigue in multiple sclerosis: a comparison of different rating scales and correlation to clinical parameters. Mult. Scler..

[bib14] Flanagan J.L., Jackson S.T. (1997). Test-Retest reliability of three Aphasia tests: performance of non-brain-damaged older adults. J. Commun. Disord..

[bib15] Genova H.M., Dobryakova E., Spirou A., Wylie G. (2019).

[bib16] Genova Helen M., Rajagopalan Venkateswaran, Deluca John, Das Abhijit, Binder Allison, Arjunan Aparna, Chiaravalloti Nancy, Wylie Glenn (2013). Examination of cognitive fatigue in multiple sclerosis using functional magnetic resonance imaging and diffusion tensor imaging. PLoS One.

[bib17] Glasser Matthew F., Sotiropoulos Stamatios N., Anthony Wilson J., Coalson Timothy S., Fischl Bruce, Andersson Jesper L., Xu Junqian, Jbabdi Saad, Webster Matthew, Polimeni Jonathan R., Van Essen David C., Jenkinson Mark (2013). The minimal preprocessing pipelines for the human connectome Project. Neuroimage.

[bib18] Habeck Christian, Rakitin Brian C., Moeller James, Scarmeas Nikolaos, Zarahn Eric, Brown Truman, Stern Yaakov (2005). An event-related FMRI Study of the neural networks underlying the encoding, maintenance, and retrieval phase in a delayed-match-to-sample task. Cogn. Brain Res..

[bib19] van Heugten C.M., Wilson B.A., Plats T. (2021). Clinical Pathways in Stroke Rehabilitation.

[bib20] Hinkle Janice L., Becker Kyra J., Kim Jong S., Choi-Kwon Smi, Saban Karen L., McNair Norma, Mead Gillian E. (2017). Poststroke fatigue: emerging evidence and approaches to management: a scientific statement for healthcare professionals from the American heart Association. Stroke.

[bib21] Kos D., Kerckhofs E., Carrea I., Verza R., Ramos M., Jansa J. (2005). Evaluation of the modified fatigue impact Scale in four different European countries. Mult. Scler..

[bib22] Laurens Kristin R., Kiehl Kent A., Liddle Peter F. (2005). A supramodal limbic-paralimbic-neocortical network supports goal-directed stimulus processing. Hum. Brain Mapp..

[bib23] Lee K.A., Hicks G., Nino-Murcia G. (1990). Validity and reliability of a Scale to assess fatigue. Psychiatry Res..

[bib24] Malloy S., Genova H., Chiaravalloti N., Deluca J., Holtzheimer P., Wylie G.R. (2021). Cognitive fatigue in traumatic brain injury : a pilot Study comparing State and trait fatigue. Brain Inj..

[bib25] Matthews Julian, Andrea Pisauro M., Jurgelis Mindaugas, Müller Tanja, Vassena Eliana, Chong Trevor T.J., Apps Matthew A.J. (2023). Computational mechanisms underlying the dynamics of physical and cognitive fatigue. Cognition.

[bib26] Motes Michael A., Rao Neena K., Shokri-Kojori Ehsan, Chiang Hsueh-Sheng, Kraut Michael A., Hart Jr John (2017). Trial-Level regressor modulation for functional magnetic resonance imaging designs requiring strict periodicity of stimulus presentations: illustrated using a Go/No-Go task. Magn. Reson. Insights.

[bib27] Müller Tanja, Apps Matthew A.J. (2019). Motivational fatigue: a neurocognitive framework for the impact of effortful exertion on subsequent motivation. Neuropsychologia.

[bib28] Mutai Hitoshi, Furukawa Tomomi, Houri Ayumi, Suzuki Akihito, Hanihara Tokiji (2017). Factors associated with multidimensional aspect of post-stroke fatigue in acute stroke period. Asian Journal of Psychiatry.

[bib29] Pedersen Annie, Almkvist Emelie, Holmegaard Lukas, Lagging Cecilia, Redfors Petra, Blomstrand Christian, Jood Katarina, Samuelsson Hans, Jern Christina (2022). Fatigue 7 years Post-Stroke: predictors and Correlated features. Acta Neurol. Scand..

[bib30] Pendlebury S.T., Klaus S.P., Mather M., de Brito M., Wharton R.M. (2015). Routine cognitive screening in older patients admitted to acute medicine: abbreviated Mental Test Score (AMTS) and subjective memory complaint versus Montreal cognitive assessment and IQCODE. Age Ageing.

[bib31] Power Jonathan D., Mitra Anish, Laumann Timothy O., Snyder Abraham Z., Schlaggar Bradley L., Petersen Steven E. (2014). Methods to detect, characterize, and remove motion artifact in resting State FMRI. Neuroimage.

[bib32] Román Cristina A.F., Deluca John, Yao Bing, Genova Helen M., Wylie Glenn R., Wylie Glenn R. (2022). Signal detection theory as a novel tool to understand cognitive fatigue in individuals with multiple sclerosis. Front. Behav. Neurosci..

[bib33] Román Cristina A.F., Wylie Glenn R., Deluca John, Yao Bing (2022). Associations of white matter and basal ganglia microstructure to cognitive fatigue rate in multiple sclerosis. Front. Neurol..

[bib34] S Wu, Barugh A., Macleod M., Mead G. (2014). Psychological associations of poststroke fatigue: a systematic review and meta-analysis. Stroke.

[bib35] Salthouse Timothy A. (1996). The processing-speed theory of adult Age differences in cognition. Psychol. Rev..

[bib36] Shenhav Amitai, Botvinick Matthew M., Cohen Jonathan D. (2013). The expected value of control: an integrative theory of anterior cingulate cortex function. Neuron.

[bib37] Shenhav Amitai, Musslick Sebastian, Lieder Falk, Kool Wouter, Griffiths Thomas L., Cohen Jonathan D., Botvinick Matthew M. (2025). Toward a rational and mechanistic account of mental effort.

[bib38] Spielberger Charles Donald, Barratt Ernest S. (1972).

[bib39] Tang W.K., Liang H.J., Chen Y.K., Chu Winnie C.W., Abrigo Jill, Mok V.C.T., Ungvari Gabor S., Wong K.S. (2013). Poststroke fatigue is associated with caudate infarcts. J. Neurol. Sci..

[bib40] Tang Wai Kwong, Chen Yang Kun, Vincent Mok, Chu Winnie C.W., Ungvari Gabor S., Ahuja Anil T., Wong Ka Sing (2010). Acute basal ganglia infarcts in poststroke fatigue: an MRI Study. J. Neurol..

[bib41] Ulrichsen K., Alnæs D., Kolskår K.K., Richard G., Sanders A.M., Dørum E.S., Ihle-Hansen H., Pedersen M.L., Tornås S., Nordvik J.E., Westlye L.T. (2020). Dissecting the cognitive phenotype of post-stroke fatigue using computerized assessment and computational modeling of sustained attention. Eur. J. Neurosci..

[bib42] Vickrey B.G., Hays R.D., Harooni R., Myers L.W., Ellison G.W. (1995). A health-related quality of life measure for multiple sclerosis. Qual. Life Res. : An International Journal of Quality of Life Aspects of Treatment, Care and Rehabilitation.

[bib43] Wylie G.R., Genova H.M., Yao B., Chiaravalotti N., Roman C.A.F., Sandroff B.M., DeLuca J. (2023). Evaluating the effects of brain injury, disease and tasks on cognitive fatigue. Sci. Rep..

[bib44] Wylie G.R., Genova H.M., Yao B., Chiaravalotti N., Roman C.A.F., Sandroff B.M., DeLuca J. (2023). Evaluating the effects of brain injury, disease and tasks on cognitive fatigue. Sci. Rep..

[bib45] Wylie G.R., Yao B., Genova H.M., Chen M.H., DeLuca J. (2020). Using functional connectivity changes associated with cognitive fatigue to delineate a fatigue network. Sci. Rep..

[bib46] Wylie Glenn R., Dobryakova E., DeLuca J., Chiaravalloti N., Essad K., Genova H. (2017). Cognitive fatigue in individuals with traumatic brain injury is associated with activation of the caudate. Sci. Rep..

[bib47] Wylie Glenn R., Dobryakova E., DeLuca J., Chiaravalloti N., Essad K., Genova H. (2017). Cognitive fatigue in individuals with traumatic brain injury is associated with activation of the caudate. Sci. Rep..

[bib48] Wylie Glenn R., Yao Bing, Sandry Joshua, Deluca John, Wylie Glenn R. (2021). Using signal detection theory to better understand cognitive fatigue. Front. Psychol..

[bib49] Van Zandvoort M.J.E., Kappelle L.J., Algra A., De Haan E.H.F. (1998). Decreased capacity for mental effort after single supratentorial lacunar infarct May affect performance in everyday life. J. Neurol. Neurosurg. Psychiatry.

