# Supplementary material for: The critical role of the basal ganglia in post-stroke fatigue: A pilot study
Source: Neuroimage Rep. 2025 Dec 18;6(1):100313. doi: 10.1016/j.ynirp.2025.100313 (PMC12771294; doi:10.1016/j.ynirp.2025.100313)

“The Critical Role of the Basal Ganglia in Post-Stroke Fatigue: A Pilot Study”

Supplemental Material

Tables

Supplementary Table 1. *The number of excluded runs across participant groups as a result of zero fatigue being reported.*

	Runs in which		
Group	VAS-F > 0	VAS-F = 0	% lost
BG-	33	0	0%
BG+	48	3	5.9%
HC	45	6	11.8%
Total	126	9	7.1%

Figures

Supplementary Figure 1. Examples unit amplitude and amplitude modulated regressors used to model task activation and cognitive fatigue (CF)-related activation, respectively. Both regressors were included in the model. The regressor of unit amplitude (panel A) models the response that is stable across blocks; the amplitude modulated regressor (panel B) models the response that is modulated by CF. Panel C shows the entire design matrix with the regressors for the three blocks on the left and the amplitude modulated regressors on the right. There are two pairs of amplitude modulated regressors: one for blocks where the response window was short and one for blocks where the response window was long. The plots in panels A and B correspond to the last two columns of the design matrix.

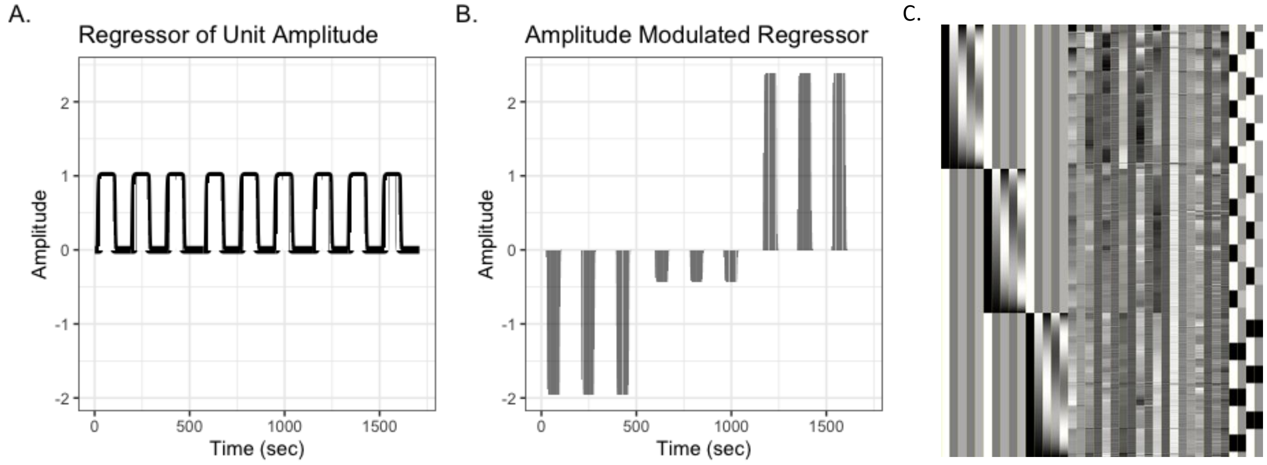

Supplementary Figure 2. Individual lesion maps for participants with basal ganglia damage (BG+ group) overlaid on a standard brain template (MNI152 2mm brain).

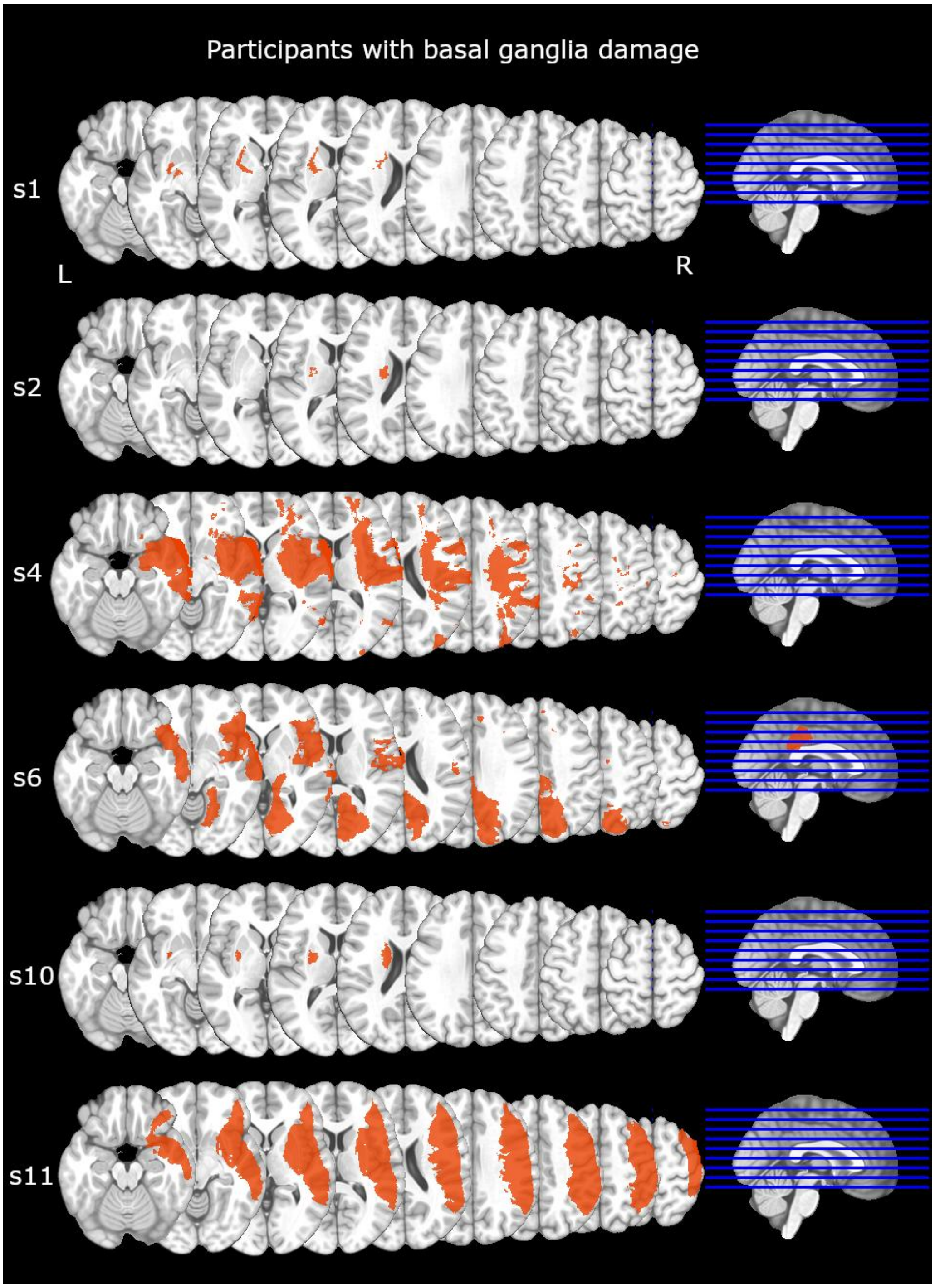

Supplementary Figure 3. Individual lesion maps for participants without basal ganglia damage (BG- group) overlaid on a standard brain template (MNI152 2mm brain).

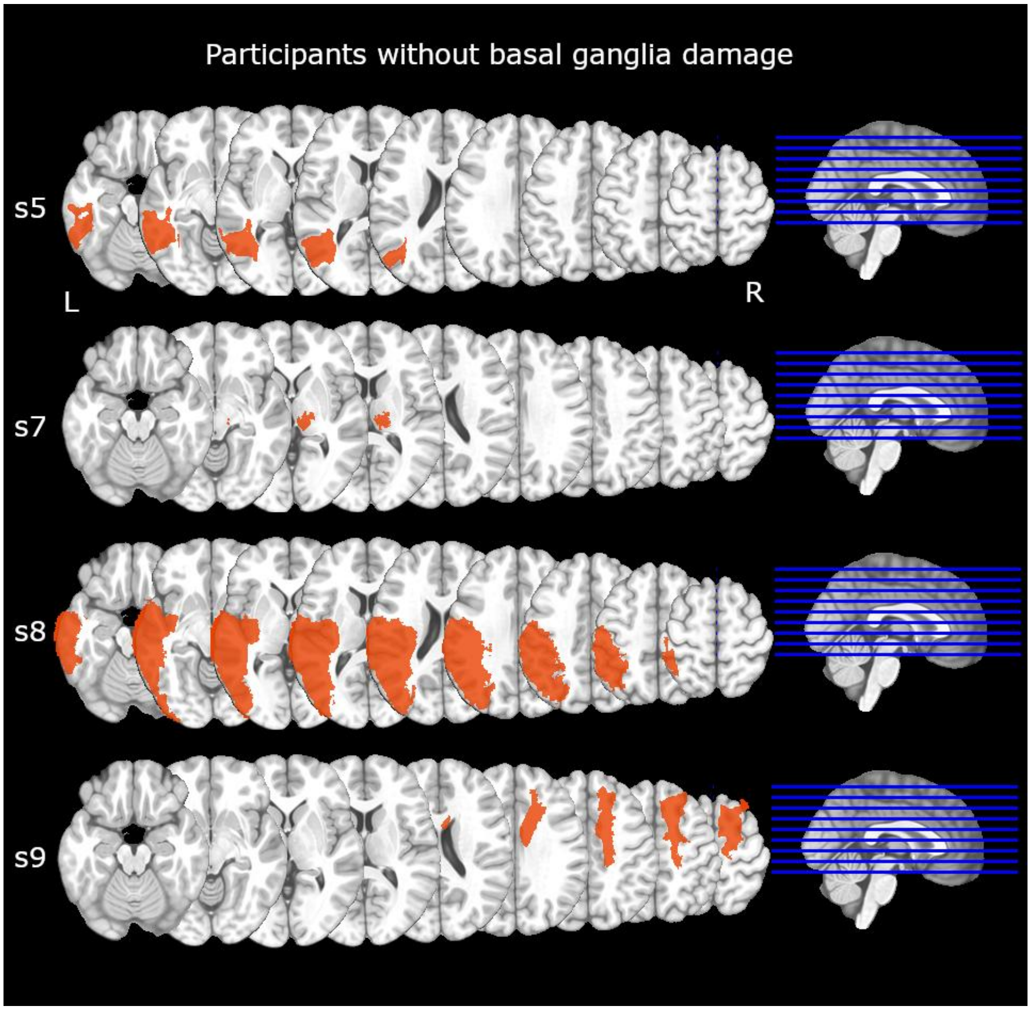

Supplementary Figure 4. Brain areas activated by the modified letter comparison (mLC) task across the three participant groups.

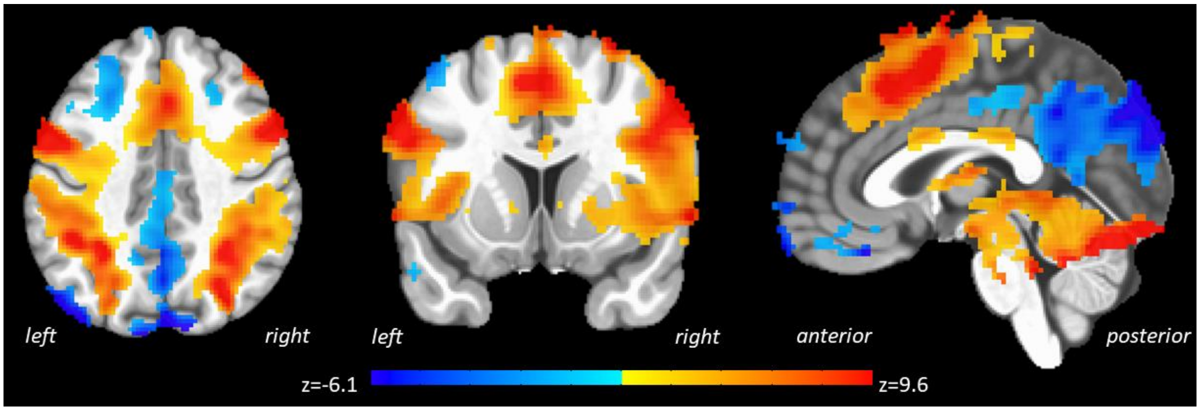

Supplement: Multimedia component 1 [file mmc1.pdf]
